# NGS-Based Application for Routine Non-Invasive Pre-Implantation Genetic Assessment in IVF

**DOI:** 10.3390/ijms22052443

**Published:** 2021-02-28

**Authors:** Katalin Gombos, Bence Gálik, Krisztina Ildikó Kalács, Krisztina Gödöny, Ákos Várnagy, Donát Alpár, József Bódis, Attila Gyenesei, Gábor L. Kovács

**Affiliations:** 1 Szentágothai Research Center, University of Pécs, Ifjúság útja 20., 7624 Pécs, Hungary; gombos.katalin@pte.hu (K.G.); galik.bence@pte.hu (B.G.); kalacs.kriszta.ildiko@gmail.com (K.I.K.); varnagy.akos@pte.hu (Á.V.); gyenesei.attila@pte.hu (A.G.); 2 Department of Laboratory Medicine, Medical School, University of Pécs, Ifjúság útja 13., 7624 Pécs, Hungary; 3 MTA-PTE Human Reproduction Scientific Research Group, University of Pécs, 7624 Pécs, Hungary; krisztina.godony@cdoki.hu (K.G.); bodis.jozsef@pte.hu (J.B.); 4 Department of Clinical Molecular Biology, Medical University of Bialystok, ul. Jana Kilinskiego 1, 15-089 Bialystok, Poland; 5 Department of Obstetrics and Gynaecology, Medical School, University of Pécs, Édesanyák útja 17, 7624 Pécs, Hungary; 6 MTA-SE Molecular Oncohematology Research Group, 1st Department of Pathology and Experimental Cancer Research, Semmelweis University, Üllői út 26., 1085 Budapest, Hungary; alpar.donat@med.semmelweis-univ.hu

**Keywords:** in vitro fertilisation, missed abortion, spent culture medium, next-generation sequencing, bioinformatic pipeline, screening

## Abstract

Although non-invasive pre-implantation genetic testing for aneuploidy (NIPGT-A) is potentially appropriate to assess chromosomal ploidy of the embryo, practical application of it in a routine IVF centre have not been started in the absence of a recommendation. Our objective in this study was to provide a comprehensive workflow for a clinically applicable strategy for NIPGT-A based on next-generation sequencing (NGS) technology with the corresponding bioinformatic pipeline. In a retrospective study, we performed NGS on spent blastocyst culture media of Day 3 embryos fertilised with intracytoplasmic sperm injection (ICSI) with quality score on morphology assessment using the blank culture media as background control. Chromosomal abnormalities were identified by an optimised bioinformatics pipeline applying copy number variation (CNV) detecting algorithm. In this study, we demonstrate a comprehensive workflow covering both wet- and dry-lab procedures supporting a clinically applicable strategy for NIPGT-A that can be carried out within 48 h, which is critical for the same-cycle blastocyst transfer. The described integrated approach of non-invasive evaluation of embryonic DNA content of the culture media can potentially supplement existing pre-implantation genetic screening methods.

## 1. Introduction

The current clinical guidelines for embryo selection related to in vitro fertilization rely on non-invasive embryo morphology assessment. For standardized and clinically applicable oocyte and embryo assessment, the grading criteria was latest updated in 2011 during the Istanbul Consensus Workshop [1]. In line up with the evolving advances of morphological evaluation [2,3] the arsenal of non-invasive methods developed based on the detection of molecular markers present in the spent culture media (SCM) of the embryo. Advances of mass spectroscopy produced sensitive proteomic methods that capable to detect altered presence of low molecular weight protein structures in the SCM-like ubiquitin [4], apolipoprotein AI [5], different hCG isoforms [6] and alpha-1 chain of the human haptoglobin [7,8] in correlation with successful implantation capacity of the blastocysts. Catabolic activity of the embryo can be monitored non-invasively by consumption of nutrients, monitoring oxidative stress or embryo respiratory rates [9,10]. Continuous development of analytical techniques like ESI-MS fingerprinting, Nano-UHPLC MS/MS, MALDI-TOF, immunoassays, microarray and NGS approaches of screening the embryo secretome, metabolome and complete cell-free nucleic acid profile from the SCM offer exceptional non-invasive way to assess embryo quality, ploidy and viability [11,12]. The use of minimal-invasive or non-invasive methods became a major factor of consideration during the genetic composition assessment of the developing embryo. Although pre-implantation genetic testing for aneuploidy (PGT-A) is integrated into many IVF programmes to achieve improvements in success [13] emerging perspectives of the non-invasive pre-implantation genetic testing for aneuploidy (NIPGT-A) [14,15] is widely welcome. Currently, growing scientific evidence emphasises the clinical applicability of SCM in NIPGT-A and the concordance of NIPGT-A with ICM biopsies [14,15,16,17,18,19]. Moreover, publications suggest that NIPGT-A has the potential to be superior to TE biopsy for aneuploidy screening [14]. However, a major pitfall of NIPGT-A is that there are many well-defined sources of DNA contamination, such as DNA from the polar bodies, cumulus cells and external, fragmented DNA contaminants. These possible contamination mechanisms have been observed by several independent study groups [14,15,16,17]. and described as key limitations of NIPGT.

To address this key limitation, the aim of our study was to develop a workflow based on next-generation sequencing (NGS) technology and the corresponding data analysis pipeline. Moreover, as a proof of principle, our goal was to develop and recommend a non-invasive screening method that can be applied within 48 h, which is a critical time window for decision making. During the development of the pipeline, we made special emphasis on minimising the noise effect of the DNA contamination. The proposed methodology was tested on spent embryonic culture media droplets of morphologically good quality embryos to avoid false positive results from disproportionate embryonic cell divisions when a higher number of embryonic cells are undergoing apoptosis due to clearance from the embryo. We also aimed to correlate NGS results with reproductive outcome.

## 2. Results

### 2.1. Sample Collection

Our embryo culture media collection completed in a period of 15 months in our IVF centre, between January 2017 and March 2018. During this period, we collected culture media of only morphologically good quality embryos for this study. Embryos were evaluated only morphologically prior to transfer and single embryo transfer (SET) was carried out in all cases. The average age of the women in the studied population was 35. In the 15-month time period during the clinical follow-up the number of total collected oocytes was 753, and the number of transferred embryos was 514. The successful pregnancy rate was 34% (184 clinically registered pregnancies) leading to the live births of 83 healthy neonates and 20 miscarriages (that counted 10.9% of the clinically registered pregnancies). We assigned all culture media samples belonging to the miscarriage group and their blank media droplets as controls and randomly selected 20 media samples and corresponding blank control media (G1 mediums) from those embryos that developed into healthy neonates for NGS analysis. We compared the culture media samples one-by-one with their corresponding blanks and the two groups according to pregnancy outcome.

### 2.2. Next-Generation Sequencing and Primary Data Analysis

**Figure 1:**
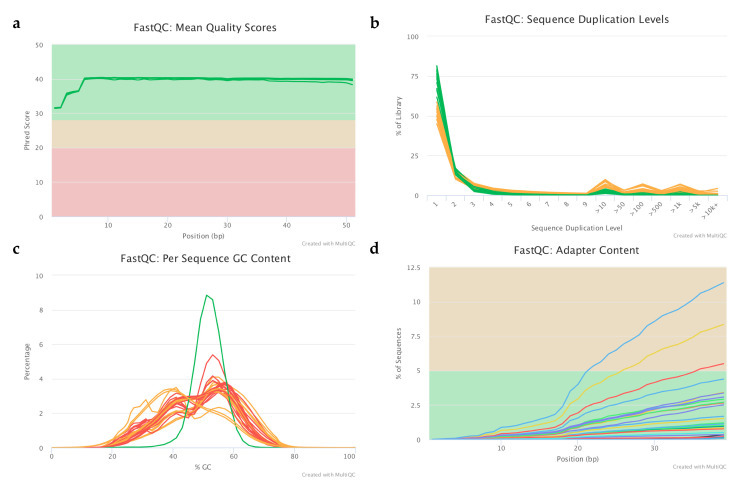
Representing plots from the raw data quality checking process with the following subfigures: (**a**) Sequence quality histogram, (**b**) Sequence duplication level, (**c**) Per sequence GC content, (**d**) Adapter content.

**Figure 2:**
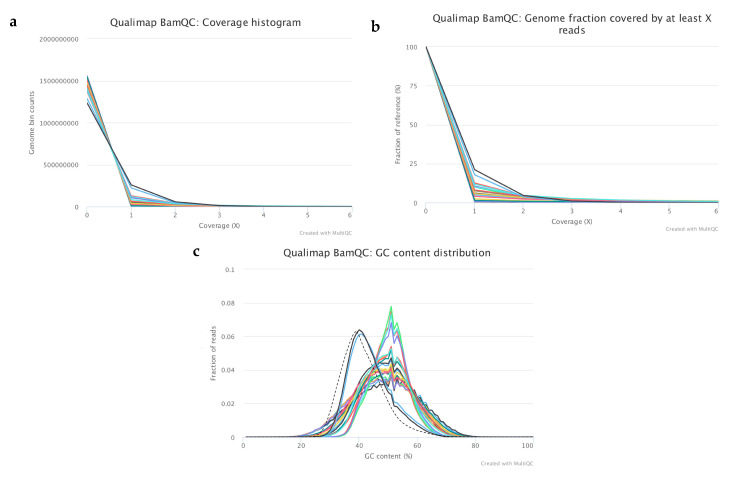
Representing plots from the analysis of mapping quality metrics of the selected samples. The subfigures represent the following results: (**a**) Coverage histogram showing the genomics bin counts with the corresponding coverage, (**b**) Cumulative coverage genome fractions showing the fraction (%) of the genome which has at least “X” coverage, (**c**) GC content distribution of the mapped reads where the dashed lines corresponds to the theoretical distribution.

After successful whole genome amplification of 28 samples, the sequencing resulted in an average 12 M reads (50 bp single end) per sample. The mean quality value across each base position in the reads were constantly above Q30, indicating high-quality sequencing reads (Figure 1a). Although the sequence duplication level was in general low for a couple of samples it was above the threshold, as depicted in yellow in Figure 2b. These samples were part of the control media and the culture media droplets of healthy neonate groups. A very similar trend can be seen in the results of GC content analysis (Figure 1c), where the GC distributions are displayed over all the sequences across the whole length and compared to a modelled normal distribution of 50% GC content (green line). The low sequencing coverage can cause unusual patterns in the GC distribution, as can be observed in Figure 1c. Finally, adapter contamination was found to be minimal for most of the samples (Figure 1d).

After mapping the filtered reads to the human reference genome (GRCh37), quality metrics were analysed, and best samples were selected (*n* = 22) for further analysis (Figure 2, Table 1). On average, 6.55% of the genome had at least 1x coverage and 0.5% had at least 5x coverage (Figure 2a,b). The rest of the genome had coverage between 0–1x across all samples. According to the mapped reads, GC distribution samples could be split into two groups (Figure 2c).

The first group consisted of only two samples—samples from the cord blood with mean GC% of 40, which is very close to the pre-calculated GC distribution for the reference genome (Figure 2c). These samples were used as controls with known copy number variations (CNVs) for data analysis optimization. The remaining samples were classified into the second group. These samples had an average of 49% mean GC content, which is slightly higher than the expected mean value (shown as a dashed line on the plot). The unusual shape of the curves in the second group is caused by the low sequencing coverage, the original DNA quality and the whole genome amplification (WGA) step. This is because the DNA was fragmented in the culture medium and the fragments origins were not uniformly distributed from the genome compared to if the DNA had been isolated from pure tissue or a small number of cells. The lower mapping percentages in the control culture media samples (35–44%) and the ratio of genomic regions that have at least 1x coverage compared to the other samples are supporting the fact there is a known fragmented DNA contamination in the culture media. Apart from these features the dataset was good for further analysis to detect CNVs.

### 2.3. Identified CNVs and Statistical Testing

**Figure 3:**
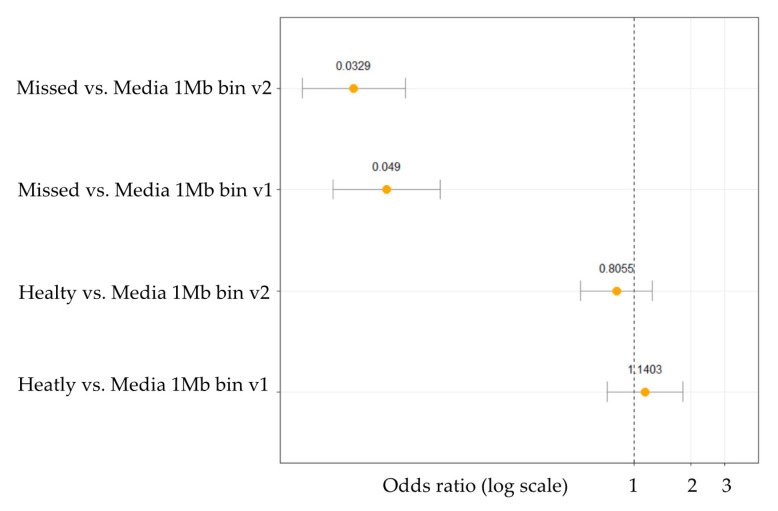
Odds ratio analysis for CNV in culture media droplets of aborted embryos (Missed) compared to control media and culture media droplets of healthy neonates (Healthy) compared to control media. Odds ratios are in log transformed scale for better visualization.

Read numbers and CNVs were counted and visualized along the whole genome according to 1 Mb bin size because of the low sequencing coverage. The number of read counts served as the basis for calculating the ploidy. The Cn.MOPS algorithm was used to predict chromosomal alterations in our dataset. After the CNV detection step odds ratios (OR) were calculated, between missed, healthy and control media groups, according to two different methods: v1—when overall CNV occurrence was counted as one main simple event on a chromosome in order to reduce the bias caused by the false positives; and v2—when every single CNV was counted separately on a chromosome. Both OR calculation methods (v1 and v2) confirmed statistically significant differences between the culture media droplets of aborted embryos (“Missed”) and the control media (“Media”) (Figure 3). In contrast, the statistical test between the culture media droplets of healthy neonates (“Healthy”) and the control media (“Media”) was not found to be significant. The main reason for this outcome could arise from the fact that the gDNA features, like fragmentation and quality, of the healthy and culture media groups were very similar, therefore we could not identify any clinically relevant alternation. Moreover, this is supported by the difference in the embryonic gDNA content and quality found in the culture media droplets of the cleavage-stage embryos that developed to healthy neonates compared to the group of embryos that were aborted. Evaluable results could be predicted only from the missed aborted embryos.

Deeper evaluation of the CNV results in the 1 Mb bins of the autosomes and comparing our results with UNIQUE database [https://www.rarechromo.org, 31 December 2020], Genetic Alliance database [https://www.geneticalliance.org.uk, 31 December 2020] and CDO database [https://chromodisorder.org, 31 December 2020] revealed 17 relevant chromosomal alterations. All of these occurred only in the aborted embryo group and were related to registered chromosomal alterations and major developmental impairments.

**Figure 4:**
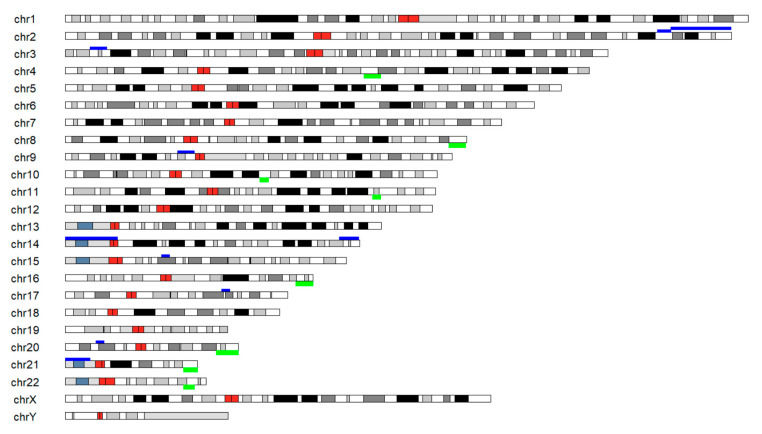
Karyogram representing clinically relevant autosomal alterations identified based on the NGS analysis of the gDNA content from the culture media of the aborted embryos. Dark red bands showing the centromeres, green bands above the chromosomes are indication gains and dark blue bands showing losses.

Table 2 lists all the identified CNVs and Figure 4 displays the variations on a karyogram. Two of the SCMs from the aborted embryo group were found to be free from recognized and clinically significant CNVs, but 9 out of the 11 SCM samples of the aborted group were positive for multiple chromosomal abnormalities.

## 3. Discussion

SCM is found to be a potentially useful liquid biopsy sample that represents embryonic genetic material [14,15,16,17,18,19,20,21,22,23,24,25,26,27,28,29,30]. Although PGT and its well-established clinical applications still have a place, advances of NIPGT-A evolve, because spent embryonic media collection does not require excess intervention and manipulation of the human embryo or any subsequent modification of the clinical routine, while it has the potential to reflect chromosomal composition of the developing embryo [14,15,16,17,18,19,20].

Some recent studies published during 2018–2021 also reported the successful amplification of cell-free DNA from spent culture media and compared their results with PGT from parallel TE biopsies [14,15,16,17,28,29,30]. These studies unanimously confirm the potential of SCM-based NIPGT-A to better reflect the actual ploidy status of the developing embryo compared to PGT of the TE biopsy. One of these studies, from Yeung et al. [28], performed NGS on embryos affected by single gene disorder and gained a few discordant results between TE and spent culture medium. By utilising the whole embryo and obtaining two biopsies from the TE and one biopsy from the ICM they obtained only 37.5% concordance rate across the biopsy series. While TE result reflects only the genetic composition of the cellular mass of the biopsy, spent blastocyst culture media is suggested to be more appropriately reflective of the overall ploidy status of the embryo at the time of collection. Comparative performance of the NIPGT-A studies is demonstrated in Table 3.

Sensitivity and specificity results of these studies were influenced by maternal DNA contamination and mosaicism. Maternal contamination resulted in misdiagnosis in embryo sex chromosome determination in a relatively high percentage (63–86%) [14,15,17]. DNA contamination of the culture media arising from culture media straight from the bottle was not detected by the work groups, not even Rubio et al. in their stratified analysis for culture media in a large multicentric study which compared different culturing conditions and culture media [16]. An earlier publication by Hammond et al. confirmed the background contamination to be extremely low by long-range and quantitative PCR methods [24], it still interfered with Y chromosome detection, in their study and a mixture of DNA fragments of sex chromosomes was detected across the batches. The major supplement in most, if not all, clinical IVF settings is HAS, which has already been proved to have high affinity for DNA. In our experiment blank media control droplets were used to identify the presence of baseline DNA contamination in the culture medium as well as contamination that may arise throughout the culture period. In most of the abovementioned publications (or in the studies published so far) NIPGT-A was performed on Day 5 blastocyst culture media. Rubio et al. extended embryo culture until Day 6 to gain higher embryonic cfDNA concentration and quality [16]. Ho et al. compared SCM from Day 3 and Day 5 embryos [29]. In their comparison, cell-free DNA from SCM of Day 3 embryos had slightly better specificity compared to Day 5 embryonic SCM (69% versus 61%) and a higher concordance rate with whole-embryo ploidy (56.3% versus 45.5%); however, Day 5 SCM performed better on overall comparison because of higher concentrations of embryonic DNA and higher ratios of generated sufficient sequence reads.

Since our goal was to complete a generally applicable non-invasive embryo selection strategy combined with same-cycle transfer, we followed the already existing clinical routine concerning IVF methods, embryo culture and transfer conditions in cases of the genetic-disease-free population of women of average age 35. In most cases in this population stratum, multiple morphologically similar, good quality oocytes are retrievable, and the cultured embryos often show equally good scores on morphological evaluation; therefore, the selection of embryos for transfer is a frequently appearing relevant clinical dilemma. We maintained the routine sequential culturing and collected spent embryonic culture media after assisted hatching (AH) on Day 3, when embryos were morphologically evaluated and moved to fresh G2 media. Considering the AH, Yeung et al. confirmed that the concentration of cell-free DNA in the culture medium was not affected by the application of AH, either on Day 3 or Day 5 [28], although NIPGT, as well as PGT, shows better results on Day 5 due to higher ICM mass of the embryos and a greater amount of leaked gDNA. In our current study we focused on the gDNA content on Day 3 of the cleavage-stage embryos’ culture media, despite the fact that gDNA content is increasing in parallel with the growing embryo mass throughout the culture time. This was because we aimed to complete our NGS workflow within 48 h, when embryo assessment results are summarised for embryo selection for SET to achieve fresh, same-cycle embryo transfer. This is important for IVF protocols that do not include embryo cryopreservation and vitrification procedures; however, our workflow can also be incorporated into the “freeze-all” or “elective frozen embryo” strategies, and the NIPGT results can also support PGT; moreover, it can be fitted into most of the currently used IVF strategies. We have to highlight that small NGS platforms like MiSeq and iSeq could be more cost effective and more suitable for real clinical practice. Moreover, there is possibility for time-lapse morphology evaluation in the time between Day 3 and Day 5 embryo culture, and additional verification of the selection decision can be gained during the sequential culturing methods. Our study design also enables the collection of multiplex data about the developing embryo, since around 5 µL of culture media of the total 20 µL is used for NGS analysis. The remainder is available for proteomic and miRNA analysis, which can also be integrated into a complex embryo assessment strategy.

## 4. Materials and Methods

### 4.1. Study Design and Workflow

To validate our workflow a total of 40 spent culture media of Day 3 embryos fertilised with intracytoplasmic sperm injection (ICSI) and presenting good quality scores on morphology assessment were collected prospectively in the Assisted Reproduction Unit, Department of Obstetrics and Gynaecology, University of Pecs, Hungary. The work described here was approved by the Committee of Human Reproduction, National Science Council of Hungary: 5273-3-2012/HER, later superset by Public Health Officer Hungarian Government Office in Baranya County: BAR/006/58-2/2014.) The research related to human use has been complied with all the relevant national regulations, institutional policies and in accordance with the tenets of the Helsinki Declaration.

After the registered pregnancy outcome, spent culture media samples and corresponding blank culture media were sequenced on an Illumina NGS HiSeq 4000 platform for copy number variation (CNV) detection.

**Figure 5:**
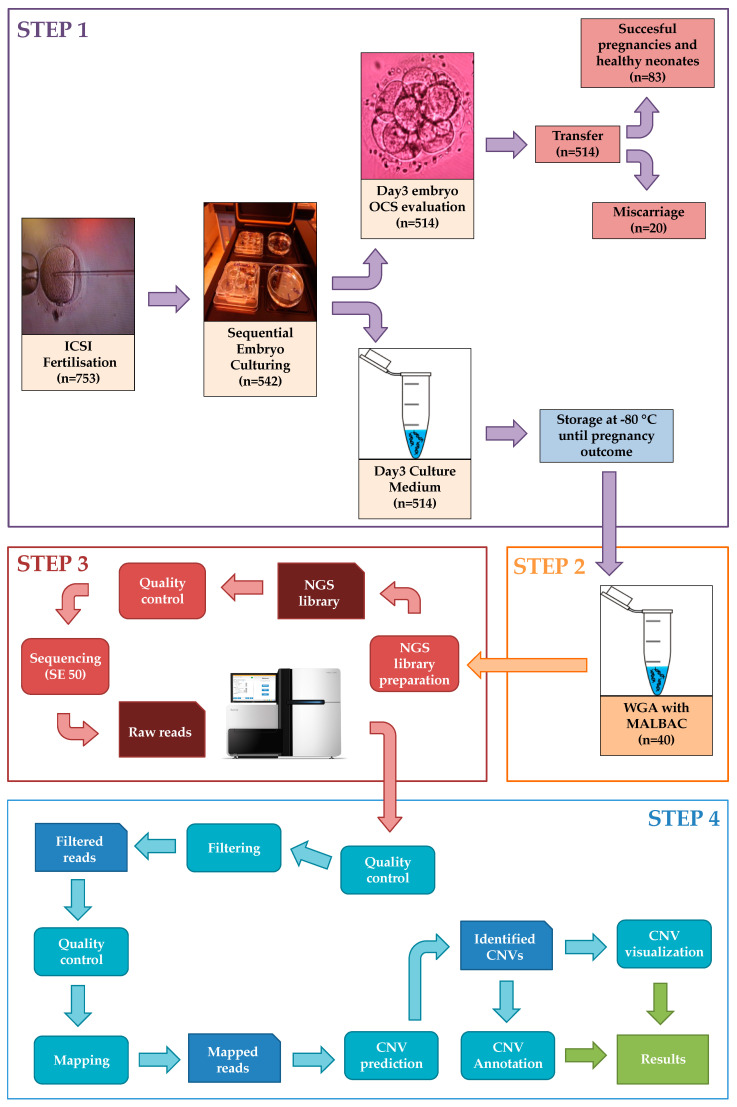
Representation of the entire workflow with all four main steps including Step 1: IVF procedure and sample collection, Step 2: Whole genome amplification. Step 3: Next-generation sequencing and Step 4: Bioinformatics analysis.

The developed comprehensive workflow shows the entire clinical procedure of IVF, the embryo culture and the wet-lab handling and dry-lab bioinformatics steps of sample processing (Figure 5). The following sections briefly describe the main steps of our proposed workflow applied to the 40 selected samples.

### 4.2. Step 1: IVF Procedure and Sample Collection

The oocytes selected for ICSI were denuded carefully with hyaluronidase and assessed for maturity. Only metaphase II oocytes (*n* = 753), identified by the presence of the first polar body, were chosen for fertilisation. Intracytoplasmic sperm injection was performed 3–6 h after oocyte recovery in a bicarbonate-buffered medium (G-IVF, Vitrolife, Gothenburg, Sweden). Fertilisation was assessed 24 h later in G-1 v5 medium (Vitrolife) supplemented with human serum albumin (HSA; Vitrolife) in 5 mg/mL concentration. Embryos (*n* = 542) were cultured following a sequential culture protocol in total 40 µL culture medium and moved to fresh medium droplets on Day 3 (*n* = 514) and 20 out of 40 µL of the spent medium was collected and stored at –80 °C for NGS analysis (*n* = 514). As negative control, we collected the same amount of blastocyst culture medium that had not been used for embryo culture. These blank media control drops were collected from the same LOT of medium and HSA. All collected samples were frozen immediately in liquid nitrogen and stored at −80 °C until subjected to whole-genome amplification and NGS library preparation. From the collected media droplets, we selected spent medium samples from embryos that were found to fulfil good composite score on the ‘optimised criteria system’ (OCS) evaluation [7]. This is a morphological evaluation system that adopts and further optimises the Istanbul consensus. According to this scoring, on the 3rd day those cleavage-stage embryos with high blastomeric number (7 or more), that fully symmetric position of the blastomeres and fragmented cell rate below 10% were assigned to the ‘good’ category.

Selected embryo morphology parameters and parental gynaecological characteristics are summarized in Table 4. After registration of pregnancy outcome in 184 cases, all spent embryo culture media samples were used for the downstream laboratory analysis from the miscarriage group (Group 0, *n* = 20). From the routinely collected culture media of transferred cleavage-stage embryos that developed to healthy neonates (*n* = 83), a matching number of 20 were randomly selected for NGS to permit group comparison and denoted as Group 1.

Spent cleavage-stage embryonic culture media samples were handled carefully to prevent media cross-contamination, and pipette tips were changed between sample collection and transfer of each media droplet. Five μL from the total of 20 μL of medium from each embryo was transferred into RNase–DNase-free PCR tubes containing 5 μL cell lysis buffer (Yikon Genomics, Beijing, China).

### 4.3. Step 2: Whole-Genome Amplification

The multiple annealing and looping-based amplification (MALBAC) WGA method was used to amplify DNA from the culture medium samples as well as from the blank media droplets, following the manufacturer’s protocol (Catalogue no. YK001B; Yikon Genomics, Beijing, China). Concentration of the whole-genome amplified products was assessed using the Qubit 2.0 fluorometric quantitation system (Life Technologies, Carlsbad, CA, USA).

### 4.4. Step 3: Next-Generation Sequencing

After the WGA step, due to low sample quality, only 28 out of 40 samples were selected for the next process. Since the concentration of DNA in the culture media is very low it makes it difficult to prepare it for sequencing. NGS libraries were prepared from 50 ng input material using the Nextera DNA Library Preparation Kit (Illumina, San Diego, CA, USA) with Nextera DNA Combinatorial Dual Indices. Briefly, DNA was fragmented, cleaned and amplified, followed by a second bead clean-up. After quality control, individual libraries were diluted, equimolarly pooled, and sequenced by Illumina HiSeq 4000 utilising patterned flow cell technology, with 50 bp single-read configuration. Selecting the right sequencing platform depends on the number of samples. The raw sequencing data was uploaded to the European Nucleotide Archive (https://www.ebi.ac.uk/ena, Primary Accession: PRJEB38821, Secondary Accession: ERP122272, 31 December 2020). In real clinical practice a smaller sequencing instrument developed for clinical applications, such as MiSeq or iSeq, would be more practical and cost efficient to fulfil the requirements.

### 4.5. Step 4: Bioinformatics Analysis

#### 4.5.1. Raw Data Quality Control and Filtering

During data pre-processing, overall quality metrics for raw sequencing reads were checked using FastQC v0.11.5 [31]. Based on these results the dataset was filtered, and the remaining adapters and low-quality (>Q30) sequences and 3′ tails removed using Cutadapt v1.18 and Trim_Galore v0.4.1 [32,33].

#### 4.5.2. Sequence Alignment and Mapping Quality

Next, filtered sequences were mapped to the *Homo sapiens* GRCh37 reference genome using BWA v0.7.13 aligner bwa mem algorithm, applying standard parameters [34]. BAM files were sorted and indexed by SAMtools v1.7 module [35]. Mapping quality and alignment results were summarised for each sample using QualiMap bamqc v2.2.1 [36]. MutliQC v1. [37] was run to combine mapping reports into one in order to compare much more easily the different results from each separate report. Based on the mapping quality results 22 out of 28 samples were selected for further analysis.

#### 4.5.3. CNV Identification

The read-count-based CNV prediction tool cn.MOPS v1.30.0 [38] was optimized to NIPGT-A. Telomere and centromere regions were excluded from the analysis. The read numbers were counted along the whole genome with a minimum bin size of 1 Mb because of the low sequencing coverage. A copy number gain from two to three copies results in a 50% increase in read counts, whereas a copy number loss from two copies to one results in a 50% decrease in read counts. Results could be exported in various formats, such as tabular or a more widely accepted VCF format.

#### 4.5.4. Statistics for CNV Analysis

In order to validate the statistical significance of the identified CNVs, ORs were calculated with 95% confidence intervals using the epi.2by2 function from the epiR R programming package [39]. Two counting methods of CNV events were applied. First, CNVs were counted separately as simple events. Second, all events in one chromosome were merged into one large event. Applying the latter method, we could reduce the false positive CNVs that result from the low sequencing coverage. Results were visualised using the ggplot2 R package [40].

#### 4.5.5. Functional Analysis

In the downstream analysis the identified alterations were functionally annotated by UNIQUE database [https://www.rarechromo.org, 31 December 2020], Genetic Alliance database [https://www.geneticalliance.org.uk, 31 December 2020] and CDO database [https://chromodisorder.org, 31 December 2020].

## 5. Conclusions

In this paper we proposed a workflow which combines low gDNA input based NGS application and downstream bioinformatic analyses in order to identify CNVs from the culture media droplets in a non-invasive way. The workflow can be carried out within 48 h which suits the same-cycle transfer. The proposed method was appropriate to handle the contaminating DNA problem at a very early developmental stage. Even if all the short and low-quality DNA fragments originating from contamination could not be removed by the wet-lab or the dry-lab part from further analysis, the applied sample preparation and bioinformatics techniques helped to indicate whether a sample may carry any chromosomal abnormalities. Carrying out additional downstream level analysis was challenging, mainly because of the low coverage, but the algorithm was sufficiently sensitive to detect reliable CNVs if the sample was good according to all quality metrics. Consistent with previous work using the MALBAC WGA method, the success rate of amplification was 95–96% [14,30].

We are aware of the fact that our study includes limitations that arise from the sample size and the lack of comparison of spent blastocyst culture media with corresponding TE and ICM NGS analysis in order to accurately describe embryonic chromosomal composition. This was due to ethical regulation of our IVF centre, which limits all invasiveness during embryo culture, but some reports published previously in this field covered this gap and confirmed the spent embryo culture media NIPGT-A to reflect, and in studies by Huang et al. Shitara et al. even outperform PGS for aneuploidy [14,15,16,17,18,19,20,21,22,23,30]. Another shortfall of our experiment is the applied assisted hatching that we routinely apply to open zona pellucida (ZP), thereby our method cannot be considered as fully non-invasive. Recent multicentric study by Rubio et al. confirmed that effective cfDNA NGS analysis can be performed when SCM is collected from embryos with intact ZP [16].

The advantage of our experiment is the complete clinical follow-up until pregnancy. Therefore, we were able to fix the endpoint of our analysis at live birth, which has not been described in the literature. The results were obtained from 542 ICSI-fertilised embryos, which were successfully transferred. The pregnancy rate was 34% for these embryos. Culture media of all embryos that suffered miscarriages and a matched number of culture media droplets of embryos from the healthy neonate group were analysed. After identification of the DNA fraction related to the cultured embryo, we found that human embryos that showed competence for blastocyst development and successful pregnancy were different in their culture media gDNA content compared to that of embryos that aborted after successful implantation. In particular, analysis of DNA profiles of Day 3 spent media demonstrated that higher gDNA copy number is associated with impaired intrauterine development and indicated miscarriage outcomes, while low gDNA of embryonic origin in the culture medium was found to be characteristic of healthy pregnancy and live birth. As our NGS analysis permitted deep CNV evaluation, chromosomal compositions of the embryos were also detected. We found clinically significant autosomal ploidy alterations only among the aborted embryos—this affected 75% of them. In some cases, the chromosomal ploidy aberration was found to be multiple, which can be irreconcilable with healthy embryonic development and embryonic viability.

## Figures and Tables

**Table 1:** Mapping quality metrics of the selected control media samples (c.), culture media droplets from embryos of miscarriage (0), culture media droplets of healthy neonates (1), cord blood sample with known CNVs (2).

Sample Name	Group	Avg. GC	≥1X	≥5X	Median Coverage	% Aligned
G1_plus_HSA1	c	49%	0.9%	0.3%	0.0X	40.9%
G1_plus_HSA2	c	49%	0.5%	0.2%	0.0X	39.7%
G1_plus_HSA4	c	47%	0.8%	0.2%	0.0X	35.1%
G1_plus_HSA5	c	48%	0.7%	0.3%	0.0X	36.2%
G1_plus_HSA6	c	48%	0.8%	0.3%	0.0X	44.2%
7567_1A	0	50%	10.6%	0.4%	0.0X	91.9%
7567_1B	0	48%	4.0%	1.1%	0.0X	76.0%
7010_1A	0	49%	1.4%	0.4%	0.0X	41.7%
7010_1B	0	50%	12.6%	0.4%	0.0X	96.2%
7301_1A	0	50%	11.9%	0.4%	0.0X	95.2%
7301_1B	0	50%	5.0%	1.0%	0.0X	84.7%
7316_1A	0	49%	6.1%	1.0%	0.0X	86.4%
7316_1B	0	50%	9.9%	0.3%	0.0X	95.7%
7370_1B	0	50%	7.5%	0.9%	0.0X	87.4%
6341_4B	1	49%	1.5%	0.4%	0.0X	40.4%
6341_4C	1	50%	2.4%	0.5%	0.0X	47.9%
7793_1A	1	49%	5.7%	1.3%	0.0X	83.1%
7793_1B	1	50%	7.8%	1.1%	0.0X	87.8%
7938_1A	1	50%	8.1%	1.1%	0.0X	88.8%
7938_1C	1	49%	9.9%	1.1%	0.0X	92.2%
A7Down	2	44%	17.8%	0.0%	0.0X	98.0%
A8Down	2	44%	21.1%	0.0%	0.0X	98.0%

**Table 2:** Chromosomal alterations found in missed aborted embryos and listed in human genetic databases (UNIQUE, Genetic Alliance and CDO).

Chromosomal Location	Type of Alteration	Function
2q35	deletion	XRCC5 gene inactivation- defect in DNA repair function
2q37	2.3-2.4 mb deletion	IGFBP2 inactivation
3p25.3-p25.1	deletion	miR-885 inactivation, impaired differentiation
4p16.3-p16.1	duplication	CNV identified with chromosomal microarray in individuals with developmental disabilities or congenital anomalies (ISCA)
8q24.3	duplication	myc proto-oncogene gene desert in GWAS studies
9p12-p11.2	deletion	ANKRD20A3 gene inactivation syndromic hydrocephalus due to diffuse hyperplasia of choroid plexus, glioma
10q22.1	duplication	COLl13A1 frameshift with pathogenic interpretation (ClinVar)
11q23.1-23.3	duplication	Beckwith-Wiedemann syndrome
14q31.1-q31.3	deletion	autosomal dominant disorder (HPPD)involving hypertelorism and deafness
14q32.2-q32.33	deletion	FOXG1 inactivation, impaired development and structural brain abnormalities
15q13.3	deletion	MTMR10, FAN1 frameshift associated with karyomegalic interstitial nephritis
16q23.3-24.3	duplication	APRT, FOXC2 indel, adenine phosphoribosyl transferase deficiency, disichtiasis lymphoedema syndrome
17q22-p23.2	deletion	Ateleiotic dwarfism, isolated growth hormone deficiency
20p12.2-p12.1	deletion	JAG1 related Alagille syndrome
20q13.31-q13.33	duplication	PKC1, phosphoenolpyruvate carboxikinase deficiency
21q22.3	duplication	RIPK4, PCNT popliteal pterygum syndrome, lethal type
21p13-p11.2	deletion	short arm loss monosomy
22q13.2-13.31	duplication	SCO2 cardioencephalomyopathy due to cytochrome c oxidase deficiency, fatal

**Table 3:** Comparative summary result of NIPGS studies using SCM, (a) Yeung et al., (b) Huang et al., (c) Xu et al., (d) Vera-Rodriguez et al., (e) Ho et al., (f) Shitara et al., (g) Rubio et al. (multicentric).

Performance	a (*n* = 116)	b (*n* = 48)	c (*n* = 42)	d (*n* = 71)	e (*n* = 41)	f (*n* = 20)	g (*n* = 1301)
sensitivity	81.6%	100%	88.2%	46.5%	80%	100%	76.5–91.3%
specificity	48.3%	80%	84%	75%	61%	87.5%	64.7–93.3%
positive predictive value	82. 6%	91.7%	79%	90.9%	47%	88.9%	65.1–92%
negative predictive value	46.7%	100%	91.3%	20.7%	88%	100%	65.2–94%
concordance for embryo ploidy	-	93.8%	-	-	56.3%	100%	70.1–77.3%
concordance for chromosome CN	-	83.3%	-	-	-	56.3%	67.7%
concordance for TE result	73.3%	-	83.4%	34.4%	-	66.7%	72.5–86.3%

**Table 4:** Embryo morphology parameters and parental gynaecological characteristics.

	Healthy Neonate (Group 1)	Miscarriage (Group 0)
Number of embryonic culture media samples sequenced	20	20
**ICCS Scoring parameters of D3 embryos**	**group 1**	**group 0**
average blastomere number	8.2	8.6
fragmentation by volume	<10%	<10%
blastomere symmetry	full	full
**Clinical characteristics**	**group 1**	**group 0**
female average age	35.18	34.74
cause of infertility -tubal factor	27.27	22.5
cause of infertility male factor	45.45	42.5
cause of infertility -other	27.27	25
basal FSH cc (IU/µL)	7.63	7.2
previous miscarriage	0	0
oocyte collected	9.3	8.6
available embryos for culture	2.5	2.5

## Data Availability

The raw sequencing data is available at the European Nucleotide Archive (https://www.ebi.ac.uk/ena, Primary Accession: PRJEB38821, Secondary Accession: ERP122272).
